# Deep Learning and Minimally Invasive Endoscopy: Automatic Classification of Pleomorphic Gastric Lesions in Capsule Endoscopy

**DOI:** 10.14309/ctg.0000000000000609

**Published:** 2023-07-03

**Authors:** Miguel Mascarenhas, Francisco Mendes, Tiago Ribeiro, João Afonso, Pedro Cardoso, Miguel Martins, Hélder Cardoso, Patrícia Andrade, João Ferreira, Miguel Mascarenhas Saraiva, Guilherme Macedo

**Affiliations:** 1 Precision Medicine Unit, Department of Gastroenterology, São João University Hospital, Alameda Professor Hernâni Monteiro, Porto, Portugal;; 2 WGO Gastroenterology and Hepatology Training Center, Porto, Portugal;; 3 Faculty of Medicine of the University of Porto, Alameda Professor Hernâni Monteiro, Porto, Portugal;; 4 Department of Mechanical Engineering, Faculty of Engineering of the University of Porto, Porto, Portugal;; 5 Digestive Artificial Intelligence Development, Porto, Portugal;; 6 ManopH Gastroenterology Clinic, Porto, Portugal.

**Keywords:** artificial intelligence, capsule endoscopy, deep learning

## Abstract

**INTRODUCTION:**

Capsule endoscopy (CE) is a minimally invasive examination for evaluating the gastrointestinal tract. However, its diagnostic yield for detecting gastric lesions is suboptimal. Convolutional neural networks (CNNs) are artificial intelligence models with great performance for image analysis. Nonetheless, their role in gastric evaluation by wireless CE (WCE) has not been explored.

**METHODS:**

Our group developed a CNN-based algorithm for the automatic classification of pleomorphic gastric lesions, including vascular lesions (angiectasia, varices, and red spots), protruding lesions, ulcers, and erosions. A total of 12,918 gastric images from 3 different CE devices (PillCam Crohn's; PillCam SB3; OMOM HD CE system) were used from the construction of the CNN: 1,407 from protruding lesions; 994 from ulcers and erosions; 822 from vascular lesions; and 2,851 from hematic residues and the remaining images from normal mucosa. The images were divided into a training (split for three-fold cross-validation) and validation data set. The model's output was compared with a consensus classification by 2 WCE-experienced gastroenterologists. The network's performance was evaluated by its sensitivity, specificity, accuracy, positive predictive value and negative predictive value, and area under the precision-recall curve.

**RESULTS:**

The trained CNN had a 97.4% sensitivity; 95.9% specificity; and positive predictive value and negative predictive value of 95.0% and 97.8%, respectively, for gastric lesions, with 96.6% overall accuracy. The CNN had an image processing time of 115 images per second.

**DISCUSSION:**

Our group developed, for the first time, a CNN capable of automatically detecting pleomorphic gastric lesions in both small bowel and colon CE devices.

## INTRODUCTION

The detection and treatment of multiple gastric lesions is of uttermost importance. Conventional esophagogastroduodenoscopy (EGD) is the current standard of care for gastric evaluation, either in a screening setting or in patients with upper gastrointestinal (GI) symptoms, given its ease in identifying and treating gastric lesions. However, upper endoscopy is an invasive examination, with a non-neglectable risk of perforation, bleeding, infection, or even cardiopulmonary adverse events (1). Furthermore, EGD can be uncomfortable, and the use of sedation techniques during the examination can increase costs (2) related to the procedure itself and result in loss of working days by patients.

**Figure 1.**
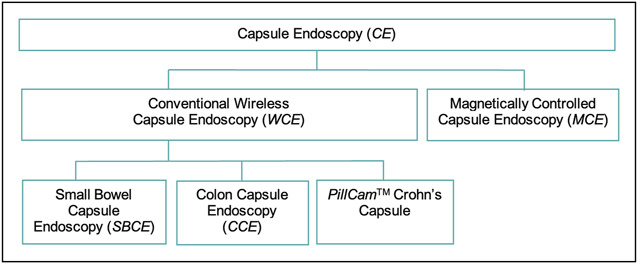
Different types of capsule endoscopies that are discussed in this article.

Capsule endoscopy (CE) is a minimally invasive examination that allows the entire visualization of the GI tract (3), with each capsule type having its own characteristics (Figure 1). Recently, it has emerged as an alternative for conventional EGD in the evaluation of the upper GI tract, especially the small bowel (4,5). With the development of colon CE (CCE), CE-based panendoscopy is an important discussion matter (6), with minimally invasive evaluation of the entire GI tract. However, CE diagnostic performance for gastric lesions is suboptimal, limiting its clinical use (7). The stomach’s anatomy is very different from that of the esophagus or duodenum, and its collapsed structure in the absence of insufflation makes the observation of all its surfaces difficult, especially the more proximal regions (8). CE dependence of the peristaltic movements can be a challenge when implementing this technology in the clinical setting. Besides, CE is a time-consuming examination, with reading times for a single examination ranging from 30 to 120 minutes (9).

The large number of image frames presented by CE devices favors the development of artificial intelligence (AI) tools for image analysis. Convolutional neural networks (CNNs) are a multilayer AI architecture with high performance levels for image analysis, inspired in the neural architecture of the human visual cortex, making it suitable for detection of imaging patterns (10). Recently, CNN models have revealed promising results in several fields of medicine (10–13). CE is one of the most studied fields for the development of CNN-based technologies for automatic detection of lesions and normal mucosa (14,15). Moreover, future application of AI tools might increase CE diagnostic yield while, at the same time, shorten its reading time and increase its cost-effectiveness. Nonetheless, the role of this technology in the identification of gastric lesions by using wireless CE (WCE) is yet to be explored. In this project, our group aimed to create a CNN-based system for the automatic classification of multiple gastric lesions using 3 different CE devices, focusing on vascular lesions (angiectasia, varices, and red spots), protruding lesions, ulcers, and erosions.

## METHODS

### Study design

This multicentric multidevice study was based on gastric images obtained from 3 different types of CE devices (PillCam SB3; PillCam Crohn's; and OMOM HD CE system) in 2 different centers (Centro Hospitalar Universitário São João and ManopH), comprising 5,846 CE examinations in 4,372 patients.

This project was developed in a noninterventional fashion (without intervention in the clinical management of each patient involved). This study was performed following the Declaration of Helsinki and with approval from the ethics committee of São João University Hospital/Faculty of Medicine of the University of Porto (No. CE 407/2020). Information potentially associated with the identification of the patients was omitted, and effective data anonymization for researchers involved in CNN development was assured by random number assignment for each patient. A legal team with Data Protection Officer Certification (Maastricht University) ensured the nontraceability of data and conformity with the general data protection regulation.

### CE protocol

CE procedures were conducted using 3 different CE devices: the PillCam SB3 system (Medtronic, Minneapolis, MN), the PillCam Crohn's (Medtronic), and the OMOM HD (Jinshan Science & Technology, Chongqing, Yubei, China). Images from PillCam SB3 and PillCam Crohn's CE were reviewed using PillCam software version 9 (Medtronic), whereas images from the OMOM HD device were reviewed using the Vue Smart software (Jinshan Science & Technology). Possible patient-identifying information (name, operating number, and date of procedure) was removed by image processing. After that, each extracted frame was stored and labeled with a consecutive number.

Each patient was asked to undergo bowel preparation in line with previous recommendations by the European Society of Gastrointestinal Endoscopy (16). In summary, patients were asked to follow a clear liquid diet on the day before capsule ingestion, with fasting the night before the examination. Before capsule ingestion, patients drank a bowel preparation consisting of 2 L of polyethylene glycol solution. Simethicone was used as an antifoaming agent. Domperidone 10 mg was used as a prokinetic if the capsule remained in the stomach 1 hour after ingestion (which implied image review on the data recorder worn by the patient).

### Classification of lesions

The gastric segment video was reviewed for identification of multiple gastric lesions. This included the first gastric image distal to the esophagogastric junction until the last image before the appearance of the duodenal mucosa. The whole group of gastric lesions included vascular lesions (angiectasia, varices, and red spots), protruding lesions, ulcers, and erosions. The definitions of the different lesions were adapted from classification scores used in small bowel CE (SBCE) (17). Regarding vascular lesions, red spots were defined as a punctuate (<1 mm) flat lesion with a bright red area, within the mucosal layer, without vessel appearance (18). Angiectasia was defined as a distinct reddish lesion constituting tortuous and clustered capillary dilations within the mucosal layer. Varices were defined as raised venous dilatation with a serpiginous appearance. Protruding lesions included polyps, epithelial tumors, subepithelial lesions, nodules, and venous structures (19). Mucosal erosions were defined as minimal loss of epithelial layering surrounded by normal mucosa. Ulcers were defined as depressed loss of epithelial covering, with a whitish base and surrounding swollen mucosa, with an estimated diameter of >5 mm.

Classification of the extracted images was performed by 3 gastroenterologists with CE expertise (M.M.S., H.C., and P.A.), each having read over 1,000 CE examinations before this study. The inclusion of a specific image implied the concordance of classification between at least 2 experts.

### CNN development

After evaluating all the examinations, 12,918 selected gastric images from 107 CE examinations were inserted into a CNN model with transfer learning. The full image data set had 1,407 images of protruding lesions; 994 from ulcers and erosions; 822 from vascular lesions; and 2,851 from hematic residues, with the remaining images being from normal mucosa.

**Figure 2.**
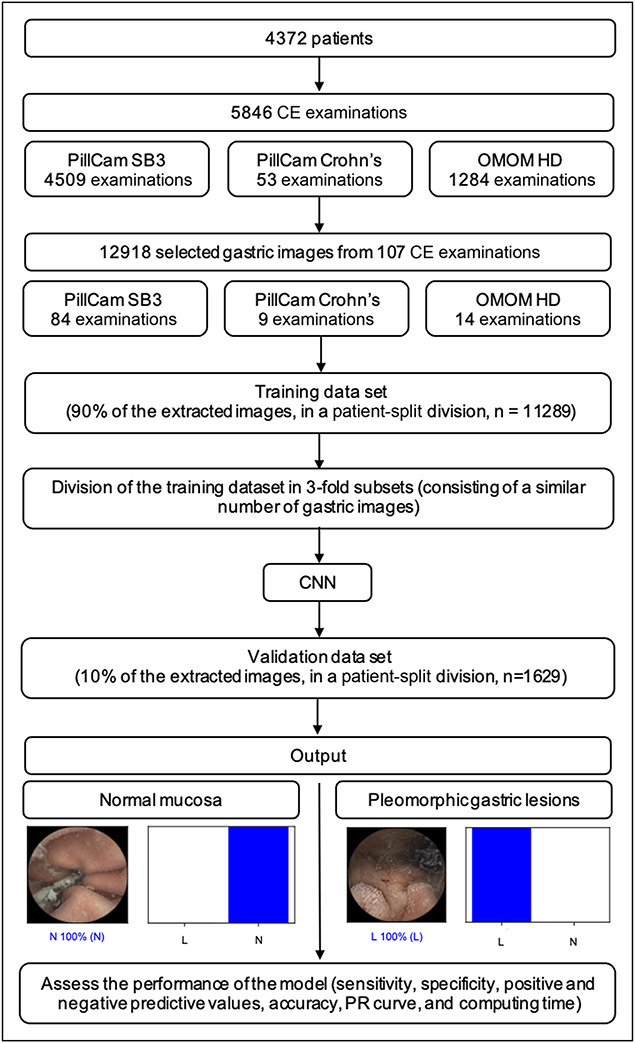
Study flowchart for the training and validation phases. CE, capsule endoscopy; CNN, convolutional neural network; L, pleomorphic gastric lesions; N, normal; PR, precision-recall.

The selected images were divided into 2 different data sets, one training data set (around 90% of the full image data set = 11,289), which was divided into 3 independent subsets, and an independent validation data set (around 10% of the full image data set = 1,629). On division, all images from a given patient were allocated to the same data set (patient-split design). The validation data set was used to evaluate the performance of the CNN model. The study design is summarized with a flowchart presented in Figure 2.

When creating the CNN, the Xception model with its weights trained on ImageNet was used. To transfer this learning to our data, the convolutional layers of the model were kept. We removed the last fully connected layers and attached fully connected layers based on the number of classes used to classify our endoscopic images.

Our group used 2 blocks, each having a fully connected layer, followed by a dropout layer of 0.25 drop rate. After these 2 blocks, a dense layer with a size defined as the number of categories to classify was added. A learning rate of 0.0001, batch size of 128, and 20 epochs were set by trial and error. We used TensorFlow 2.3 and Keras libraries to prepare the data and run the model. The analyses were performed with a computer equipped with an Intel Xeon Gold 6130 processor (Intel, Santa Clara, CA) and a NVIDIA Quadro RTX 4000 graphic processing unit (NVIDIA, Santa Clara, CA).

### Performance measures and statistical analysis

**Figure 3.**
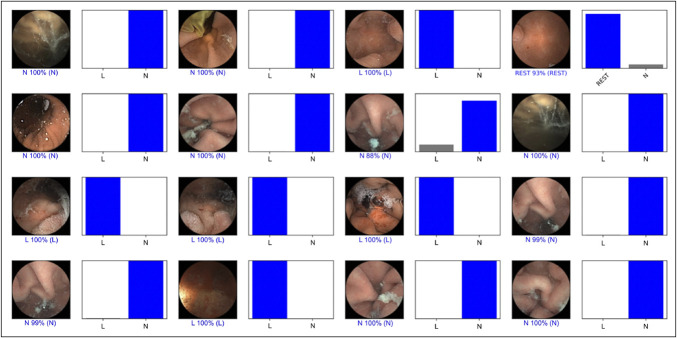
Output obtained from the application of the CNN for pleomorphic gastric lesions. The bars represent the estimated probability by the CNN model. The finding with the highest probability was outputted as the predicted classification. The blue bars represent a correct prediction, whereas the red bars represent an incorrect prediction. CNN, convolutional neural network; L, pleomorphic gastric lesions; N, normal.

**Figure 4.**
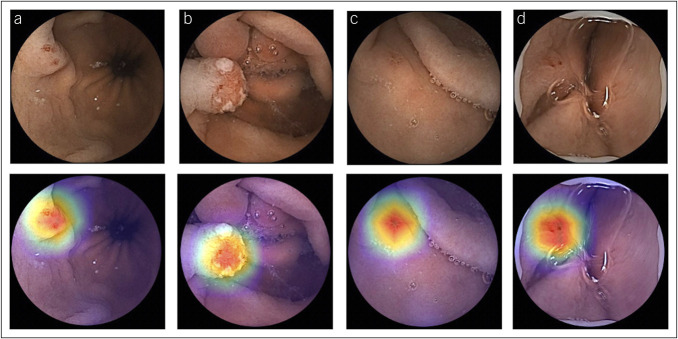
Heatmaps obtained from the application of the CNN showing pleomorphic gastric lesions as identified by the CNN. CNN, convolutional neural network; L, pleomorphic gastric lesions; N, normal.

For each image, the CNN model calculated the probability for each category (pleomorphic gastric lesions vs normal), with a given probability (Figure 3). A higher probability translated into a greater CNN prediction confidence. The software-generated heatmaps localized features that predicted a lesion probability (Figure 4). The CNN's output was compared with a consensus classification provided by 2 WCE-experienced gastroenterologists.

At the first experiment, a 3-fold cross-validation was performed, with the division of the development data set into 3 even-sized image subsets. The primary performance measures included sensitivity, specificity, positive predictive value (PPV), negative predictive value (NPV), and accuracy. These measures were represented with their means and 95% confidence intervals. The precision-recall (PR) curve and area under the precision-recall curve were used to measure the performance of the model.

The performance results from the 3-fold subsets were used to identify the better parameters of the CNN. These parameters were then applied in the validation data set, which was evaluated afterward.

Statistical analysis was performed using Sci-Kit learn version 0.22.2 (20).

## RESULTS

### Construction of the network

From a total of 5,846 CE examinations, our group developed a CNN-based model with 12,918 gastric images. Each image was evaluated by the CNN, which predicted a classification, subsequently compared with the experts' labelling. The repeated data inputs in the subset folds of the training data set allowed the adjustment of the CNN parameters. Subsequently, the fine-tuned CNN performance was evaluated in the validation data set.

### Global performance of the network

For the first analysis, a 3-fold cross-validation of the training data set was performed. The performance results of the 3-folds of the training model are presented in Table 1. Overall, the training data set had a mean sensitivity of 87.8%, specificity of 92.3%, PPV of 91.4%, and NPV of 89.2%. The mean accuracy of the model was 90.2%.

**Table 1.** Performance measures of the 3-fold cross-validation of the training data set and validation data set for detection of pleomorphic gastric lesions

—→	Sensitivity, % (CI)	Specificity, % (CI)	PPV, % (CI)	NPV, % (CI)	Accuracy, % (CI)	AUPRC
Fold 1 (n = 3,754)	81.8 (79.9–83.5)	97.8 (97.1–98.4)	97.2 (96.3–97.9)	85.3 (84.0–86.5)	90.1 (89.1–91.0)	0.93
Fold 2 (n = 3,755)	90.8 (89.4–92.1)	83.8 (82.1–85.4)	83.8 (82.4–85.2)	90.8 (89.5–91.9)	87.2 (86.0–88.2)	0.96
Fold 3 (n = 3,780)	91.0 (89.5–92.2)	95.4 (94.3–96.2)	94.7 (93.6–95.6)	92.0 (90.9–93.0)	93.2 (92.4–94.0)	0.98
Training data set mean (n = 11,289)	87.8 (86.9–88.7)	92.3 (91.6–93.0)	91.4 (90.6–92.0)	89.2 (88.5–89.9)	90.2 (89.6–90.7)	
Validation data set (n = 1,629)	97.4 (96.0–98.4)	95.9 (94.4–97.1)	95.0 (93.3–96.3)	97.8 (96.7–98.6)	96.6 (95.6–97.4)	1.00

AUPRC, area under the precision-recall curve; CI, confidence interval; NPV, negative predictive value; PPV, positive predictive value.

In the second analysis, the remaining 10% of the retrieved images were used as a validation data set for evaluation of the CNN's performance. The confusion matrix between the trained CNN and experts' classification is presented in Table 2. The CNN model identified pleomorphic gastric lesions with a sensitivity of 97.4%, specificity of 95.9%, PPV of 95.0%, NPV of 97.8%, and accuracy of 96.6% (Table 1). The model had an area under the precision-recall curve of 1.00.

**Table 2.** Confusion matrix of the automatic detection vs the experts’ classification in the validation data set of the CNN model

		Experts’ classification	
		N	L
CNN classification	N	865 (0.96)	19 (0.03)
	L	37 (0.04)	708 (0.97)

Number of cases (relative frequency).

CNN, convolutional neural network; L, pleomorphic gastric lesions; N, normal gastric images.

### Computational performance of the CNN

The CNN's image processing time of the validation data set was 115 images per second (8.7 milliseconds per frame), with a total viewing time of 14.2 seconds.

## DISCUSSION

In this proof-of-concept study, our group developed a WCE-based CNN capable of detecting multiple gastric lesions in both SBCE and CCE devices. This model demonstrated high performance levels in all the evaluated parameters, with 97.4% sensitivity, 95.9% specificity, and 96.6% global accuracy for pleomorphic gastric lesions. These results were achieved with an image processing time of 115 images per second. Furthermore, the diagnostic yield of the model was verified not only in SBCE devices but also in CCE, with 3 different types of CE devices. Thereby, our group reckons that the development of AI-powered WCE might change the landscape regarding the classification of pleomorphic gastric lesions (protuberant, vascular, ulcers, or erosions).

Furthermore, it is important to consider some methodology points about this study. The division between training and validation data sets was based on a patient-split design. Thus, all the given images from a single patient were included in the same data set. In the development of a CNN model, the inclusion of similar images in both data sets could imply an overfitting of the model (because the model would recognize a very similar image present in the training data set). Therefore, we believe that the risk of the model's overfitting was reduced. Moreover, a 3-fold cross-validation was performed. The data set was divided into 3 equal-sized, nonoverlapping folds, with images from a single examination allocated exclusively to one-fold. The average of these metrics across all iterations was used as an aggregate measure of the model's performance. This aggregated performance measure provides a more reliable assessment of the model's ability to classify new, unseen images, increasing confidence in the model's generalizability. In addition, our group used PR curves instead of receiver-operating characteristic (ROC) curves for the model evaluation. In cases of data imbalance in a certain variable, ROC curves are known for being excessively optimistic in the evaluation of a model/biomarker performance (21), with PR curves being a proved alternative (22). In our study, this was defined by a larger representation of normal gastric mucosa than gastric lesions. Given our focus on determining all the lesion images, instead of identifying the commoner true negatives, which are implied in the ROC curve construction, the PR curve was then preferred.

The interoperability challenge is one of the main points of interest in the implementation of AI-based technologies in medicine (23), with the need for generalization of technology across multiple platforms and devices. The interoperability challenge has been a point of focus in the recent years in the electronic health records system (24), but a recent article by Tang et al stated interoperability between different systems to be a determinant factor for the application of AI tools in radiology (25). Therefore, the results of our work in 3 different CE devices, either in SBCE or CCE, are a proof of the interoperability of the CNN in different outsets, fundamental for its implementation in the clinical practice. This is, to our knowledge, the first CNN model capable of diagnosing pleomorphic gastric lesions in 3 different CE devices, comprising both SBCE and CCE.

Recently, the concept of WCE-based panendoscopy has been the focus of numerous studies (26). However, CE (particularly CCE) is a time and resource-consuming examination with a large number of image frames produced, which is a disadvantage against conventional endoscopy evaluation. Furthermore, despite numerous studies about CNN models in small bowel and colon evaluation by WCE (9,15), there are no published articles about CNN models for gastric evaluation in WCE. Therefore, implementation of a specific gastric CNN, associated with small bowel and colon CNN, is important for increasing the diagnostic yield and cost-effectiveness of WCE-based panendoscopy, giving it an advantage over conventional endoscopy by providing simultaneous evaluation of the full length of the GI tract in a minimally invasive single examination. Our specific gastric CNN, with proven results in both SBCE and CCE devices, may contribute to reduce WCE reading times while increasing its diagnostic yield, reducing the subjective bias in image evaluation by experts. The integration and application in the clinical practice of such CNN would be pivotal for the implementation of a minimally invasive WCE-based panendoscopy.

Nonetheless, there are intrinsic limitations of the WCE to be considered. WCE is performed without air insufflation and stomach cavity distention, and the devices' movement depends only on abdominal peristalsis. This results in a significant reduction in stomach surface visualization, mainly the more proximal part and the fundus. The evaluation of gastric lesions by CE has regained a renewed interest with the development of magnetic controlled CE (MCE), a subtype of CE with good performance in evaluating gastric lesions, even in asymptomatic individuals (27,28). Xia et al (29) were the first to develop a CNN-based model for the detection of multiple gastric lesions and applied it to MCE. Their model focused on identifying 7 categories of gastric images (erosions, polyps, ulcers, submucosal tumors, xanthomas, normal mucosa, and invalid images). The CNN-based model had 96.2% sensitivity, 76.2% specificity, 16.0% PPV, 99.7% NPV, and 77.1% accuracy, with an area under the curve of 0.84. Although this study represents a significant breakthrough in the application of AI to minimally invasive techniques of gastric inspection, MCE is limited to very few research centers, contrary to widely available conventional WCE. In addition, MCE implies a learning curve for procedure performance (30). Besides, there is, to our knowledge, a lack of comparative studies between WCE and MCE diagnostic yield in the evaluation of gastric lesions. Whereas MCE may overcome the peristalsis dependence, it is still affected by the absence of stomach distention, with reduction in the proximal stomach visibility. In fact, a small study of MCE in healthy volunteers by Liao et al showed that, despite visualization of the more distal stomach regions by WCE in 100% of the patients, visualization of the gastric cardia and the fundus was only achieved in 82.4% and 85.3% of the patients, respectively (31). Moreover, our model showed better performance marks in all the evaluated parameters, including image processing. Thus, our group believes that the better performance of our model, combined with the worldwide availability of WCE devices, will contribute to make the generalization of this model more feasible. In addition, MCE primary focus is not a panendoscopic evaluation of the GI tract but a minimally invasive alternative for conventional EGD.

Our group developed the first CNN model for gastric lesion evaluation by SBCE and CCE with high sensitivity, specificity, accuracy, and image processing capacity. However, despite the promising results of our model, this is only a proof-of-concept study and the first step to the application of this model to a real clinical scenario.

Nonetheless, our study has several limitations. First, this study was conducted in a retrospective manner. Therefore, in the future, larger prospective multicentric studies are needed for assessment of the clinical utility of this tool. Furthermore, our results were based on still images, requiring studies with real-time WCE videos in the future.

In conclusion, the use of CNN models in clinical practice can become the standard of care in only a few years. In the gastroenterology field, optimization of WCE examinations with CNN-based technologies has been evolving recently, but the role of these systems for detection of gastric lesions in conventional WCE has not been explored yet.

Our CNN system was the first, to our knowledge, to detect gastric lesions with high accuracy and sensitivity, with excellent imaging processing times in SBCE and CCE devices. The application of these systems in clinical practice will favor the cost-effectiveness of WCE in a panendoscopy evaluation, with an associated standardization of the classification and reduction of the reading time of the examination. Larger multicentric prospective real-time studies are needed to confirm this proof-of-concept study.

## CONFLICTS OF INTEREST

**Guarantor of the article:** Miguel Mascarenhas Saraiva, MD, PhD.

**Specific author contributions:** M.M. and F.M.: equal contribution in study design, image extraction, drafting of the manuscript, and critical revision of the manuscript. T.R. and J.A.: bibliographic review, image extraction, and critical revision of the manuscript. P.C. and M.M.: bibliographic review, drafting of the manuscript, and critical revision of the manuscript. J.F.: construction and development of the CNN, statistical analysis, and critical revision of the manuscript. P.A., H.C., M.M.S., and G.M.: study design and critical revision of the manuscript. All authors approved the final version of the manuscript.

**Financial support:** The authors recognize NVIDIA support for the graphic unit acquisition.

**Potential competing interests:** None to report.

**Study Highlights**

**WHAT IS KNOWN**

✓ Capsule endoscopy (CE) is a minimally invasive examination for evaluating the entire gastrointestinal tract.
✓ The diagnostic yield of CE for gastric lesions is suboptimal.

**WHAT IS NEW HERE**

✓ Our group developed a convolutional neural network-based model for the classification of pleomorphic gastric lesions.
✓ The model’s diagnostic yield was verified in both small bowel and colon CE devices.
✓ Artificial intelligence algorithms could increase the diagnostic yield of capsule panendoscopy.
